# Black Si-doped TiO_2_ nanotube photoanode for high-efficiency photoelectrochemical water splitting

**DOI:** 10.1039/c8ra00021b

**Published:** 2018-02-02

**Authors:** Zhenbiao Dong, Dongyan Ding, Ting Li, Congqin Ning

**Affiliations:** Institute of Electronic Materials and Technology, School of Materials Science and Engineering, Shanghai Jiao Tong University Shanghai 200240 China dyding@sjtu.edu.cn; State Key Laboratory of High Performance Ceramics and Superfine Microstructure, Shanghai Institute of Ceramics, Chinese Academy of Sciences Shanghai 200050 China

## Abstract

Black Si-doped TiO_2_ (Ti–Si–O) nanotubes were fabricated through Zn metal reduction of the Ti–Si–O nanotubes on Ti–Si alloy in an argon atmosphere. The nanotubes were used as a photoanode for photoelectrochemical (PEC) water splitting. Both Si element and Ti^3+^/oxygen vacancies were introduced into the black Ti–Si–O nanotubes, which improved optical absorption and facilitated the separation of the photogenerated electron–hole pairs. The photoconversion efficiency could reach 1.22%, which was 7.18 times the efficiency of undoped TiO_2_. It demonstrated that a Si element and Ti^3+^/oxygen vacancy co-doping strategy could offer an effective method for fabricating a high-performance TiO_2_-based nanostructure photoanode for improving PEC water splitting.

## Abstract 2

Black Si-doped TiO_2_ (Ti–Si–O) nanotubes were fabricated through Zn metal reduction of the Ti–Si–O nanotubes on Ti–Si alloy in an argon atmosphere.

## Graphical abstract

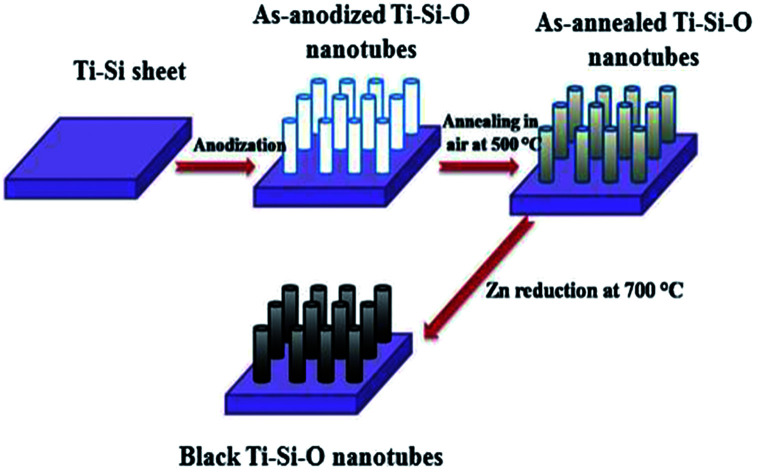

## 1 Introduction

PEC solar-driven water splitting hydrogen production based on semiconductor photocatalysts has been regarded as a promising method to decrease fossil fuel consumption and solve environmental problems.^1^ Among many photocatalysts, titanium dioxide (TiO_2_) has attracted wide interest since the first report of PEC water splitting in 1972 because of its low cost, favorable conduction band edge and chemical inertness.^2–5^ However, it suffers from two major drawbacks. One is the short diffusion paths of the charge carriers, and another is its large band gap. This leads to rapid recombination of photoinduced electrons–holes and little absorption in the visible light region.^6,7^ Therefore, great efforts have been made to improve the photoelectrical properties through ion doping,^8,9^ narrow band gap semiconductor coupling,^10,11^ noble metal deposition,^12,13^ quantum dot sensitization, *etc.*^14,15^

In recent years, black TiO_2_ through self-doped Ti^3+^ and/or oxygen vacancies has emerged as an effective approach to improve the photocatalytic performance.^16^ The defect states in bulk TiO_2_ can change the transmission path of photogenerated carriers and promote the separation-transfer process of electrons and holes. The semiconductor conductivity and charge transport were effectively promoted due to the introduction of Ti^3+^.^17^ Therefore, the photocatalytic efficiency of black TiO_2_ can be improved. In addition, Si element doping has been also proved to be a feasible method to improve the photoelectrical performance of TiO_2_.^18–20^ To date, several synthesis methods such as hydrogen thermal treatment,^17^ hydrogen plasma,^21^ chemical reduction,^22,23^ chemical oxidation,^24^ electrochemical reduction^25^ and anodization–annealing^26^ have been proposed to prepare Ti^3+^ self-doped TiO_2_-based photocatalysts. Meanwhile, according to published literatures, Si-doped TiO_2_ photocatalysts could be synthesized by chemical vapor deposition,^20^ hydrothermal process,^27^ or anodizing Ti plates in HF/Na_2_SiF_6_ solution.^28^ However, most of these methods are generally impractical for large-scale commercial production due to high cost or complicated conditions. Up to now, there was no literature report about the preparation of Si element and Ti^3+^ co-doping TiO_2_ nanotubes on Ti–Si alloy for PEC water splitting.

Inspired by thermal reduction reaction and ions-doping modification methods,^8,9,22^ we fabricated black Ti–Si–O nanotubes through Zn-reduction of the as-annealed Ti–Si–O nanotubes on Ti–Si alloy. The microstructures, optical property and the PEC water splitting properties were investigated. Electronic structures were also calculated, and possible mechanism of the black Ti–Si–O for PEC water splitting was proposed. As expected, the black Ti–Si–O nanotubes photoanode co-doped with Si element and Ti^3+^ exhibited remarkable PEC water splitting properties in comparison with undoped TiO_2_.

## 2 Experimental

### 2.1 Preparation of the black Ti–Si–O nanotubes

**Fig. 1:**
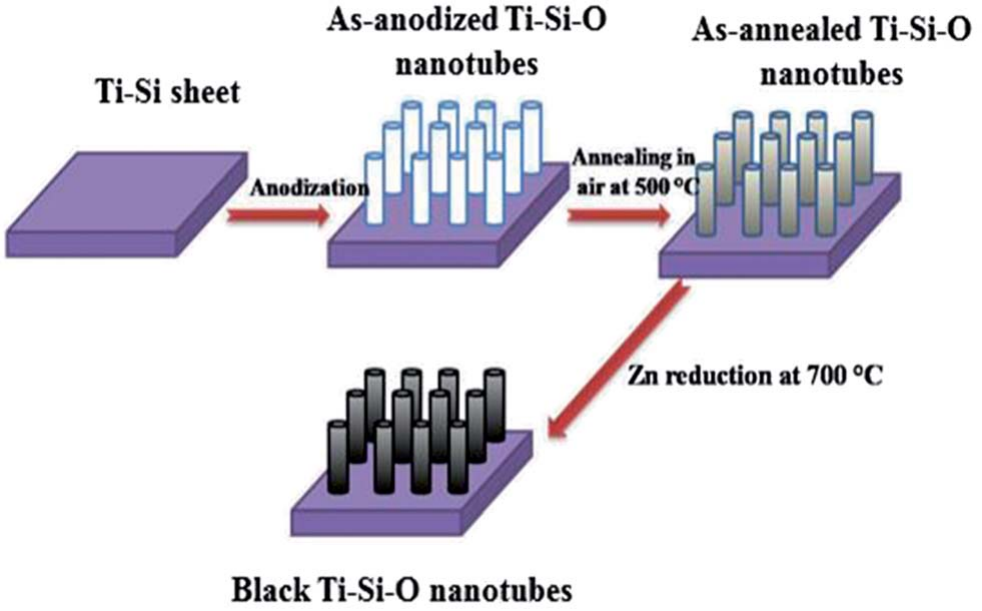
Schematic diagram fabrication processes of the black Ti–Si–O nanotubes.

A schematic illustration of fabrication procedures for black Ti–Si–O nanotubes is shown in Fig. 1. Before the fabrication of black Ti–Si–O nanotubes, Ti–Si–O nanotube arrays were obtained through anodizing Ti–5 wt% Si alloy (Ti–5Si) at 40 V for 20 min in ethylene glycol-based electrolytes (mixed by 0.5 wt% NH_4_F and 3 vol% H_2_O in ethylene glycol). The as-anodized Ti–Si–O sample was heat-treated at 500 °C in an electrical furnace for 2 h to induce crystalline phase. The as-annealed Ti–Si–O sample and Zn particles were put in two ceramic boats and sent into tube furnace. Finally, black Ti–Si–O nanotubes were fabricated through Zn reduction of the as-annealed Ti–Si–O samples at 700 °C in argon atmosphere for 4 h. For reference, undoped TiO_2_, Si-doped TiO_2_ and black undoped TiO_2_ nanotubes were also fabricated.

### 2.2 Material characterization

Morphology of the black Ti–Si–O nanotubes was observed with a scanning electron microscope (SEM, FEISIRION 200). Typical nanotubes morphology and corresponding element composition were characterized with a transmission electron microscope (TEM, JEOL JEM-2100) and the attached energy-dispersive X-ray (EDX) spectrometer. Phase composition and crystal structure was analyzed by Raman spectra with a 532 nm laser excitation wavelength on a thermal dispersive spectrometer. X-ray photoemission spectra (XPS) were obtained on a Kratos Axis Ultra DLD system to investigate the surface chemical states. The optical absorption property was evaluated by UV-visible absorption spectra measured on a Lambda 750S UV-vis spectrophotometer.

### 2.3 PEC measurement

The PEC water splitting properties were measured based on an electrochemical working station (CHI Instruments, model CHI660C). The conventional three-electrode system consists of black Ti–Si–O nanotubes working electrode, Pt foil counter electrode and Ag/AgCl reference electrode was put into 1.0 M KOH electrolyte solution during PEC measurement. A xenon-lamp with calibrated intensity 100 mW cm^−2^ provides the simulated light source. The photocurrent response was recorded by linear sweep voltammetry curve and amperometric *I*–*t* curve. Electrochemical impedance spectroscopy was obtained under dark condition at 0 V *vs.* Ag/AgCl. Mott–Schottky plots measurement was also conducted at 1000 Hz in dark.

### 2.4 Electronic structure calculation

Electronic structure calculation was carried out using the Cambridge Serial Total Energy Package (CASTEP) module based on the density functional theory (DFT) first-principles.^18^ Si atom and oxygen vacancy were built based on 2 × 2 × 1 anatase TiO_2_ supercell as the computational model of black Ti–Si–O. During the simulation calculations, the Perdew–Burke–Ernzerhof (PBE) exchange–correlation function under generalized gradient approximation (GGA) was adopted.^19^ The cut-off energy set as 340 eV, with Brillouin zone *k*-point mesh of 3 × 3 × 3. The convergence criterion of electronic self-consistent energy and atom residual force was 5 × 10^−5^ eV per atom and 10^−3^ eV Å^−1^, respectively. Electronic structure calculation was performed under these conditions after finishing geometry optimization.

## 3 Results and discussion

**Fig. 2:**
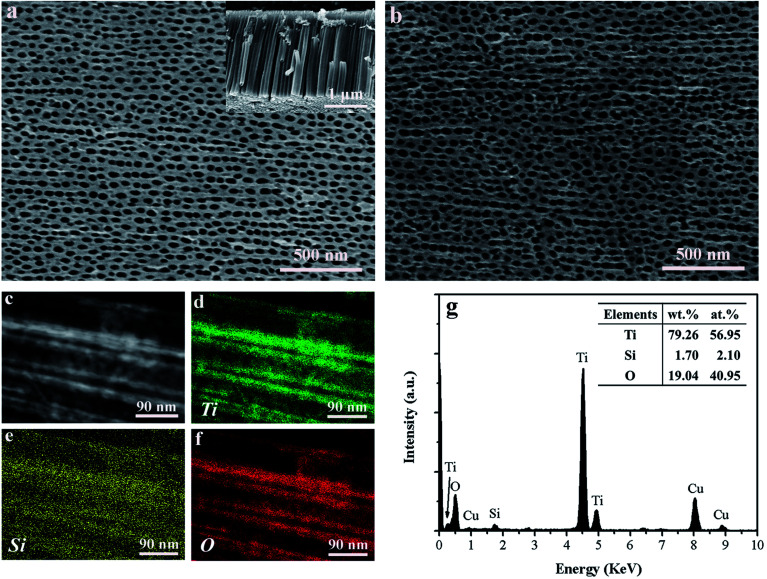
(a) Surface morphology of the Ti–Si–O nanotubes, the inset shows cross-sectional view of Ti–Si–O nanotubes. (b) Top view SEM image of the black Ti–Si–O nanotubes. (c) Typical TEM image of the black Ti–Si–O nanotubes and corresponding EDX mapping images of (d) Ti, (e) Si and (f) O. (g) EDS pattern and chemical composition of the black Ti–Si–O nanotubes.

Fig. 2a shows top view SEM image of the Ti–Si–O nanotubes. The average nanotubes diameter was around 55 nm. From cross-sectional view SEM image (inset of Fig. 2a), it presented regular and well-aligned nanotube arrays with average nanotubes length of about 2.0 μm. The SEM image of black Ti–Si–O nanotubes is shown in Fig. 2b. It still kept nanotubular morphology after Zn reduction at 700 °C, demonstrating that the Ti–Si–O nanotubes had good thermal stability. Typical TEM image of the black Ti–Si–O nanotubes can be found in Fig. 2c. Corresponding EDX mapping images and composition are shown in Fig. 2d–g. It revealed that Ti, Si and O elements were distributed in the nanotubes, suggesting that Si element existed in the black Ti–Si–O nanotubes.

**Fig. 3:**
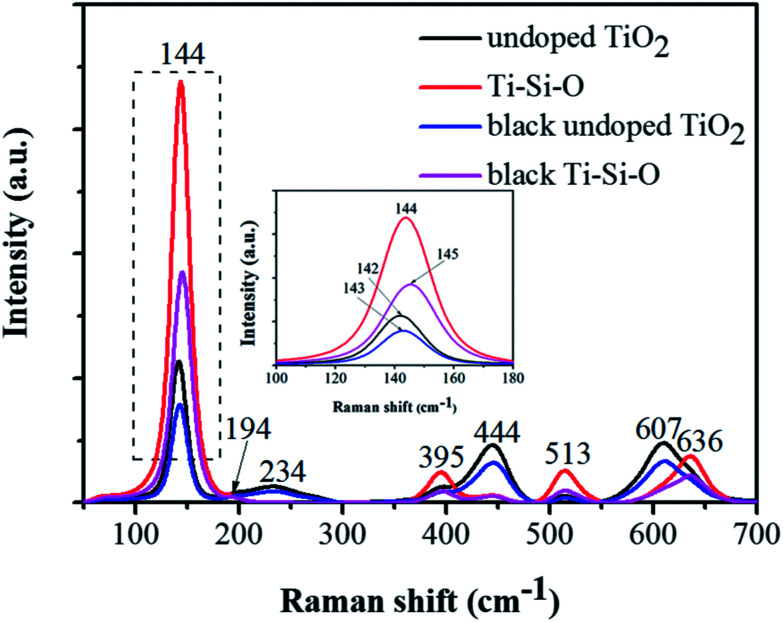
Raman spectra of the undoped TiO_2_, Ti–Si–O, black undoped TiO_2_ and black Ti–Si–O nanotubes. The inset of this figure shows enlargement peaks in the dashed line region.

Raman spectra were obtained to analyze the phase structure of the black Ti–Si–O nanotubes (Fig. 3). The undoped, Si-doped and black undoped TiO_2_ nanotubes fabricated at 700 °C were also characterized in order to identify the difference of their phase structures. For undoped TiO_2_, typical Raman shift (cm^−1^) detected at around 142 (B_1g_), 234 (E_g_), 444 (E_g_) and 607 (A_1g_) corresponded to rutile peaks,^29^ while the Raman modes detected at around 395 (B_1g_) and 513 (A_1g_) corresponded to anatase TiO_2_.^29^ Thus, we confirmed that rutile phase mainly existed in the undoped TiO_2_. It shifted to a slight higher Raman shift and no impurity phase was found for the Si-doped system, which was caused by Si-doping. Also, the intensity of rutile Raman peaks (at around 234 (E_g_), 444 (E_g_) and 607 (A_1g_) cm^−1^) decreased and it mainly consisted of anatase phase from the remnant typical anatase Raman peaks.^29^ This indicated that Si-doping could suppress the phase transformation from anatase to rutile, the result was in agreement with previous report.^30^ After Zn reduction, no obvious phase composition altered for the black undoped TiO_2_ and black Ti–Si–O. However, it is worth noting that the strongest E_g_ mode at 142 cm^−1^ of undoped TiO_2_ and 144 cm^−1^ of Ti–Si–O showed a blue shift after Zn reduction. In addition, the peaks intensity decreased with broadened full-width half-maximum (FWHM) (inset of Fig. 3). This could be analyzed by the induced oxygen vacancies according to previous studies, as it could cause crystal domain size and non-stoichiometry effects.^22,31^

**Fig. 4:**
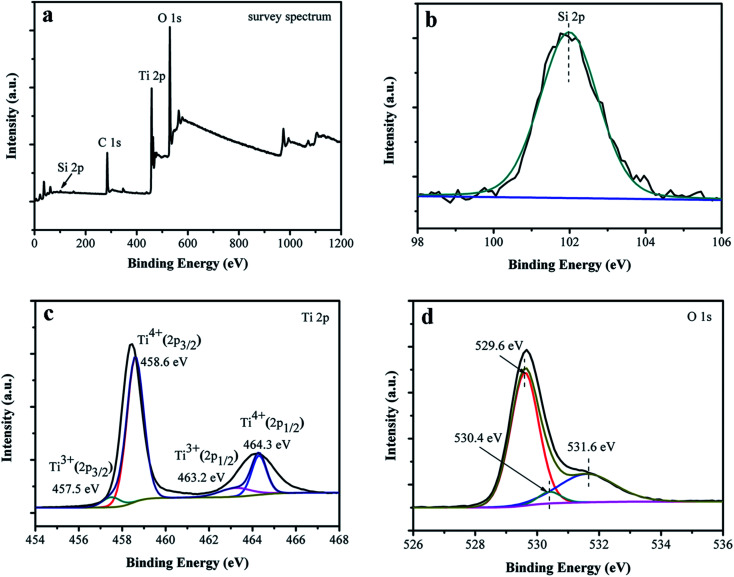
XPS results of the black Ti–Si–O nanotubes. (a) Full spectrum. High resolution spectra of (b) Si 2p, (c) Ti 3d and (d) O 1s.

Composition and chemical states of surface elements of the black Ti–Si–O nanotubes were investigated with XPS. Fig. 4a shows full XPS spectrum of the black Ti–Si–O nanotubes. Si, Ti and O elements were detected, suggesting that they existed in the black Ti–Si–O nanotubes. In addition, no evident Zn 2p peaks were found from the full spectrum, suggesting that Zn metal may only help to reduce Ti–Si–O nanotubes and little Zn residues left on the surface of black Ti–Si–O nanotubes. This result was in accordance with our TEM EDS analysis, in which no obvious Zn signals were detected. High resolution spectra of the Si 2p, Ti 3d and O 1s of the sample surface were recorded (Fig. 4b–d). The binding energy located at around 101.9 eV for Si 2p (Fig. 4b). It was lower than the Si 2p binding energy of SiO_2_ (103.4 eV), indicating effective Si-doping due to the electronegativity difference between Ti (1.54) and Si (1.90).^20,32,33^ The Ti 2p high resolution spectrum of black Ti–Si–O nanotubes was shown in Fig. 4c. Two peaks at around 458.6 eV and 464.3 eV were assigned to Ti^4+^ 2p_3/2_ and Ti^4+^ 2p_1/2_ species, respectively. While another two peaks at 457.5 eV and 463.2 eV were attributed to Ti^3+^ states.^34–36^ The O 1s spectrum was shown in Fig. 4d. It could be fitted into Ti–O (529.6 eV), Si–O (530.4 eV) and oxygen vacancy (531.6 eV), respectively.^36,37^ The result further confirmed that oxygen vacancies formed together with Ti^3+^ states, *i.e.*, both of them coexisted in the black Ti–Si–O nanotubes.

**Fig. 5:**
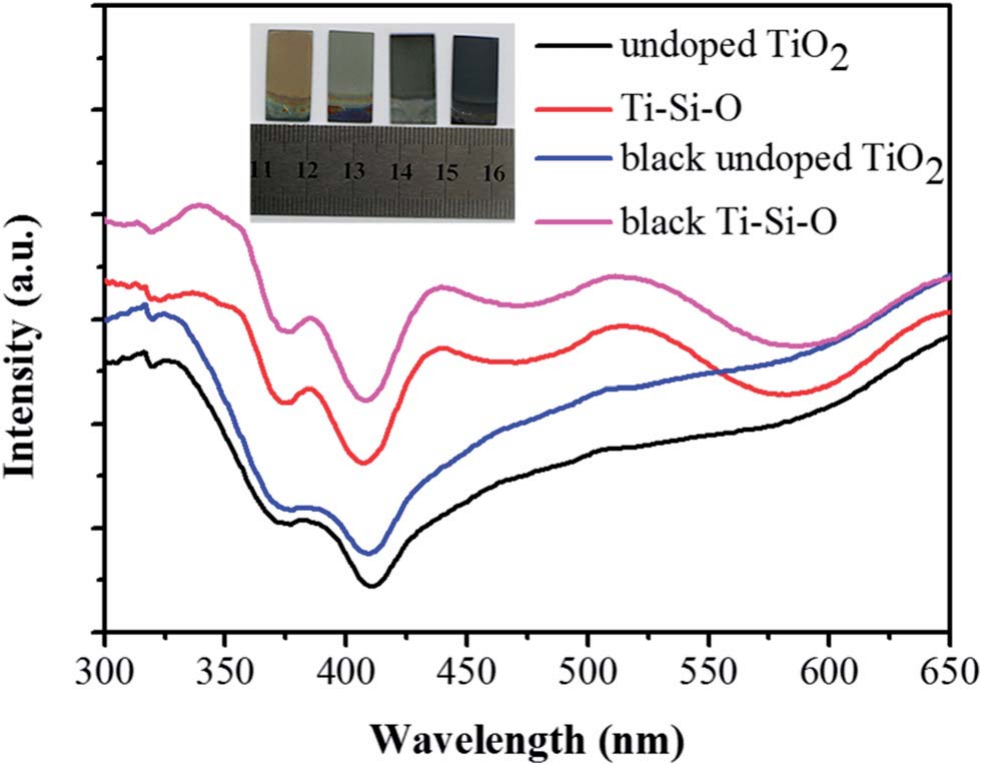
UV-vis absorption spectra of the undoped TiO_2_, Ti–Si–O, black undoped TiO_2_ and black Ti–Si–O nanotubes. The inset shows digital photographs of different TiO_2_ samples.

UV-vis absorption spectra of different TiO_2_ samples were measured to study the optical absorption property. As shown in Fig. 5, the absorption edge of undoped TiO_2_ showed a red shift after Si-doping. After Zn reduction, the absorption intensity of black undoped TiO_2_ intensified and the absorption edge appeared red shift compared to undoped TiO_2_. Meanwhile, black Ti–Si–O nanotubes presented improved absorption intensity and the absorption edge showed a further red-shift compared to those of undoped TiO_2_, Ti–Si–O and black undoped TiO_2_. According to the Kubelka–Munk equation,^38^ the extended red-shift absorption edge corresponded to a narrowed band gap for the black Ti–Si–O. Surface colors of different TiO_2_ nanotubes photoanodes were also observed and the digital photographs were shown in the inset of Fig. 5. The undoped TiO_2_ and Ti–Si–O shows pale yellow and pale blue, respectively. While the black undoped TiO_2_ and black Ti–Si–O shows black color. This could also be an indirect reflection of Ti^3+^/oxygen vacancies self-doping in the black Ti–Si–O nanotubes, as it could form color center and be favorable for visible light absorption.^39,40^

**Fig. 6:**
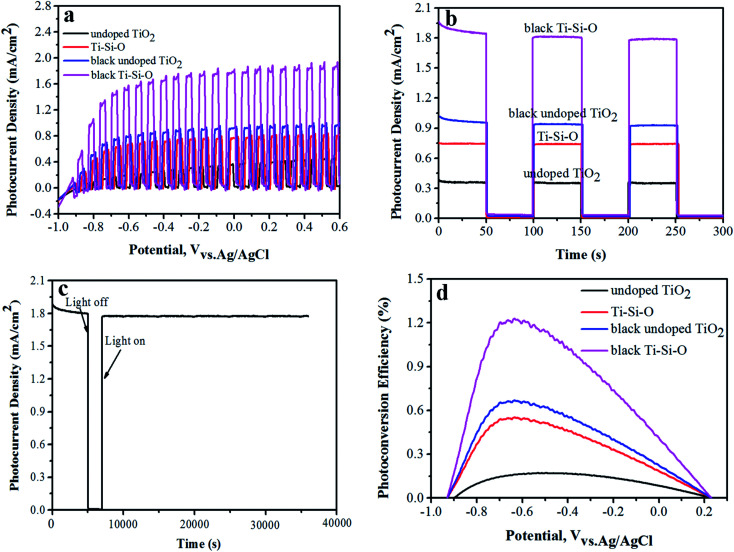
(a) Transient *I*–*V* curves and (b) potentiostatic transient *I*–*t* response of the different TiO_2_ nanotubes photoanodes. (c) PEC stability of the black Ti–Si–O nanotubes photoanode. (d) Photoconversion efficiency of the different TiO_2_ nanotubes photoanodes.

Transient linear sweep voltammetry (*I*–*V*) curve and potentiostatic transient *I*–*t* response was recorded to evaluate PEC properties of the black Ti–Si–O nanotubes photoanode. Fig. 6a and b shows transient *I*–*V* curves and *I*–*t* responses of the different TiO_2_ nanotubes photoanodes. At the same applied bias (0 V relative to the reference electrode), the photocurrent density was 0.35 mA cm^−2^ for undoped TiO_2_. It was 0.76 mA cm^−2^ for Ti–Si–O, indicating that Si-doping facilitated the separation of photogenerated carriers.^27,28^ After Zn reduction, the photocurrent density of black undoped TiO_2_ increased to 0.92 mA cm^−2^ and the black Ti–Si–O nanotubes photoanode exhibited much better photocurrent response with a remarkable photocurrent density of 1.78 mA cm^−2^. This suggested that Ti^3+^ exhibited very efficient charge separation capability of photogenerated electrons and holes. Apparently, the PEC property of the undoped TiO_2_ was greatly improved due to Si-doping and Zn reduction process. In other words, the synergistic effects of Si-doping and self-doped Ti^3+^ in the black Ti–Si–O nanotubes played a significant role in promoting the PEC water splitting performance.

Photoelectrode stability is an important indicator for PEC water splitting.^13^ It can be reflected by the change of photocurrent density *versus* irradiation time. The photocurrent density of black Ti–Si–O nanotubes photoanode under nearly 10 h continuous illumination was measured. It showed no obvious decay during long-time irradiation (Fig. 6c), suggesting that the black Ti–Si–O nanotubes photoanode exhibited good PEC stability and thus it was feasible used for high-efficiency PEC water splitting.

The solar-to-hydrogen photoconversion efficiency (*η*) meets the eqn (1):^41^

1 *η* = *I*(1.23 − *V*_RHE_)/*J*_light_

where *J*_light_ is the illumination intensity (100 mW cm^−2^), and *I* is the photocurrent density. The reversible hydrogen potential *V*_RHE_ could be obtained according to the equation of *V*_RHE_ = *V*_Ag/AgCl_ + 0.059pH + 0.199,^29^ where *V*_Ag/AgCl_ represents the applied bias, and pH value is 13.6 for the KOH electrolyte.

As shown in Fig. 6d, the black Ti–Si–O nanotubes photoanode showed a peak value of 1.22%, which was about 7.18 times that of undoped TiO_2_ (0.17%). The black Ti–Si–O nanotubes photoanodes exhibited a higher photoconversion efficiency, which was mainly caused by Ti^3+^ species. The Ti^3+^ could capture electrons effectively and prevent the photogenerated electron–hole from rapid recombination,^42,43^ which made the photon utilization rate increase. Therefore, the PEC property of the black Ti–Si–O photoanode was improved.

**Fig. 7:**
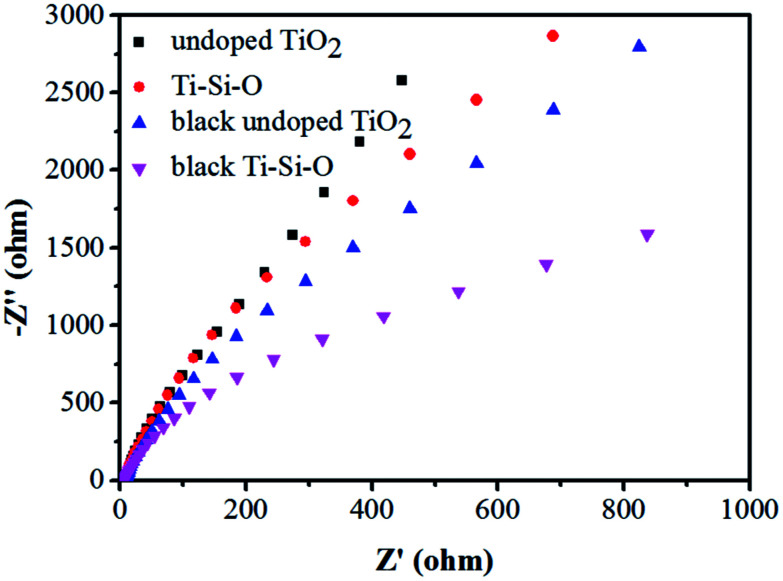
Nyquist plots of the different TiO_2_ nanotubes photoanodes.

EIS spectra of the different photoanodes were also recorded to evaluate the charge transfer characters between the semiconductor electrode and the interface. Fig. 7 shows Nyquist plots of the different photoanodes. Obviously, the black Ti–Si–O exhibited a smaller impedance arc diameter compared with undoped TiO_2_, Ti–Si–O and black undoped TiO_2_. The smaller arc diameter indicated that it was favorable for charge transfer across the interface of semiconductor electrode and electrolyte.^23^

**Fig. 8:**
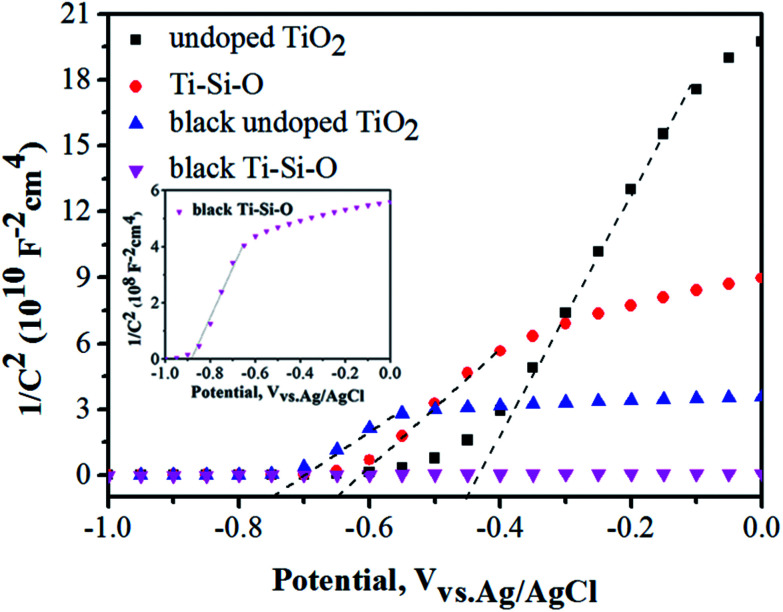
MS plots of the different TiO_2_ samples. The inset of this figure shows Mott–Schottky plots of black Ti–Si–O nanotubes photoanode.

Mott–Schottky (MS) plots of the different TiO_2_ photoanodes were measured and shown in Fig. 8. The flat band potential (*V*_FB_) and conducting type can be acquired according to eqn (2):^29,34^

2 1/*C*^2^ = (2/*e*_0_*εε*_0_*N*_d_)[(*V* − *V*_FB_) − *kT*/*e*_0_]

where *e*_0_ is the fundamental electron charge, *ε*_0_ and *ε* is the permittivity of vacuum and dielectric constant of TiO_2_, respectively. *V* is the applied potential at the electrode. As can be seen from Fig. 8, all the TiO_2_ samples present n-type semiconductor property according to positive slope in the MS plots.^42^ The acquired *V*_FB_ of undoped TiO_2_, Ti–Si–O, black undoped TiO_2_ and black Ti–Si–O nanotubes photoanodes are −0.45 V, −0.64 V, −0.75 V and −0.88 V, respectively. More negative *V*_FB_ implied more favorable charge separation and transport efficiency when used as photoelectrode.^42,44^

Meanwhile, the carrier density (*N*_d_) can be estimated by the eqn (3):^29,45^

3 *N*_d_ = (2/*e*_0_*εε*_0_)[d(1/*C*^2^)/d*V*]^−1^

A smaller slope of the linear part in MS plots corresponded to large donor density according to previous reports.^17,46^ Apparently, the black Ti–Si–O nanotubes photoanode presented a smaller slope than that of undoped TiO_2_, Ti–Si–O, black undoped TiO_2_ samples. This indicated that the as-annealed Ti–Si–O nanotubes reduced by Zn induced an increase of donor density. The donor density of black Ti–Si–O was calculated to be 1.66 × 1021 cm^−3^, which was higher than that of 2.28 × 1019 cm^−3^ for black undoped TiO_2_, 1.23 × 1019 cm^−3^ for Ti–Si–O and 6.15 × 1018 cm^−3^ for undoped TiO_2_. As a result, it could improve the conductivity and charge transport of black Ti–Si–O nanotubes photoanode. In addition, the increased donor density could raise the Fermi energy toward to conduction band, which could cause the band bending at semiconductor photoelectrode/electrolyte interface and facilitate the separation-transfer process of charge carriers.^22,23^

**Fig. 9:**
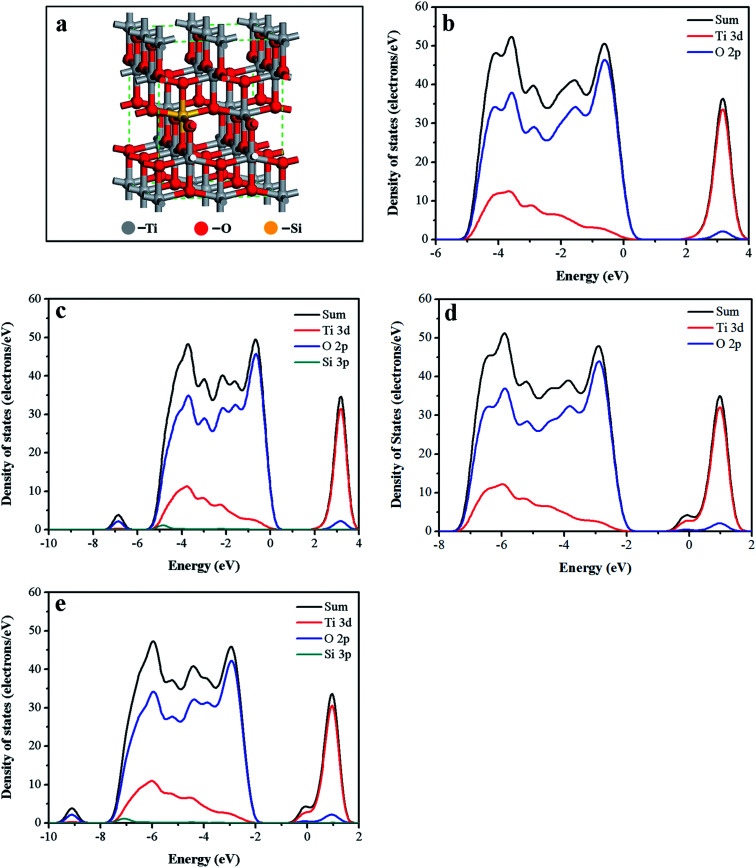
(a) The computational model of black Ti–Si–O. DOS of (b) undoped TiO_2_, (c) Ti–Si–O, (d) black undoped TiO_2_ and (e) black Ti–Si–O.

Electronic structure of the black Ti–Si–O was calculated to better understand the roles of Si-doping and Ti^3+^ self-doping. Electronic structures of the undoped TiO_2_, Ti–Si–O and black undoped TiO_2_ samples were also calculated as comparisons. Optimized computational model of the black Ti–Si–O was shown in Fig. 9a. Fig. 9b shows density of sates (DOS) of the undoped TiO_2_. Ti 3d and O 2p states constitute conduction band (CB) and valence band (VB), respectively. The calculated band gap (2.19 eV) of the undoped TiO_2_ was smaller than the experimental value due to self-shortcoming of the exchange-change function.^47,48^ No foreign levels appeared in the band gap of Ti–Si–O (Fig. 9c). But the VB was broadened due to Si doping. This facilitated separation and transportation process of the photogenerated electron–hole.^18^ Compared to undoped TiO_2_ and Ti–Si–O, the CB bottom and VB maximum of the black undoped TiO_2_ and black Ti–Si–O (Fig. 9d and e) shifted to a more negative potential. This was probably caused by Ti^3+^ self-doping, as the hybridization of Ti 3d states with O 2p and Si 3p states might be responsible for the shift of VB and CB.^40,48^ The negative shift of the energy band made the Fermi energy rise closely to conduction band. When the black Ti–Si–O nanotubes photoanode was in contact with electrolyte, the band bending degree would increase and thus enhance the separation process of the photoinduced electrons and holes. Therefore, H^+^ was prone to be reduced to H_2_ by the photogenerated electrons during PEC water splitting hydrogen evolution reaction process. In addition, for both the undoped TiO_2_ and Si-doped TiO_2_, the black process could induce the formation of miniband rising up closely below the conduction band minimum. This could result in narrowed band gap according to previous reports,^22,49^ which could enhance optical absorption shown by UV-vis absorption spectra.

**Fig. 10:**
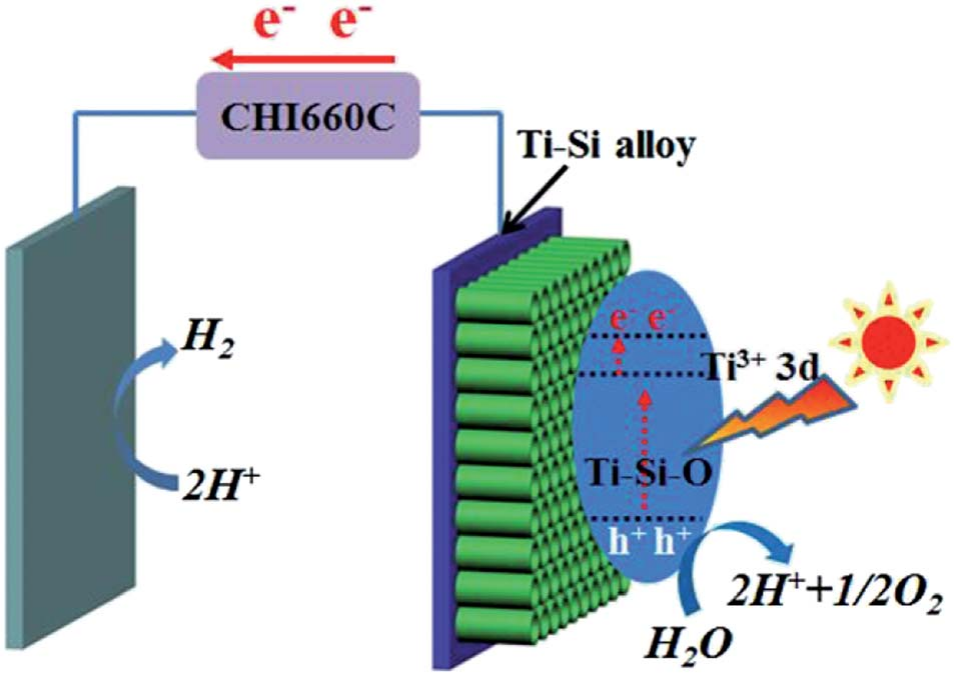
Energy diagram and charge transfer in the black Ti–Si–O nanotubes photoanode for PEC water splitting.

Based on above experimental results and analyses, possible mechanism of the black Ti–Si–O nanotubes photoanode for PEC water splitting was proposed. Energy diagram and charge transfer in the black Ti–Si–O nanotubes photoanode was illustrated in Fig. 10. Ti^3+^ 3d states were introduced into the Ti–Si–O nanotubes and thus produced donor states below the conduction band.^39^ The Ti^3+^ could form conduction band tail, which could trap photogenerated electrons and decrease the recombination rate of the charge carriers.^22,49^ Furthermore, the narrowed band gap could improve optical absorption.^31^ Moreover, Si-doping favored the separation process of the photoinduced electrons and holes.^19,50^ Once illuminated, the photogenerated holes would accumulate at the surface of the Ti–Si–O nanotubes for water oxidation (H_2_O + 2h^+^ → 2H^+^ + 1/2O_2_). Meanwhile, the photogenerated electrons quickly transferred to the cathode across the external circuit and participated in hydrogen evolution reaction (2H^+^ + 2e^−^ → H_2_). As a result, the PEC water splitting performance of black Si-doped TiO_2_ nanotubes photoanode was significantly improved.

## 4 Conclusions

In summary, black Si-doped TiO_2_ nanotubes photoanode was prepared by Zn metal reduction of the as-annealed Ti–Si–O nanotubes in argon atmosphere. Si element and Ti^3+^/oxygen vacancies were introduced into the black Ti–Si–O nanotubes, resulting in much better PEC properties than those of undoped TiO_2_ and Si-doped TiO_2_. The improved PEC performance was mainly attributed to synergistic results of enhanced optical absorption, favorable charge separation and transfer property. A higher photocurrent density of 1.78 mA cm^−2^ at 0 V *vs.* Ag/AgCl was obtained. The maximum photoconversion efficiency was 1.22%, which was about 7.18 times the photoconversion efficiency of undoped TiO_2_. We expected that these findings may contribute to develop high-performance TiO_2_-based nanostructures photoanodes for improving PEC water splitting.

## Conflicts of interest

The authors declare no competing financial interest.

## Supplementary Material
